# Solutions for Critical Raw Materials under Extreme Conditions: A Review

**DOI:** 10.3390/ma10030285

**Published:** 2017-03-13

**Authors:** Maria Luisa Grilli, Tiziano Bellezze, Ernst Gamsjäger, Antonio Rinaldi, Pavel Novak, Sebastian Balos, Radu Robert Piticescu, Maria Letizia Ruello

**Affiliations:** 1 Italian National Agency for New Technologies, Energy and Sustainable Economic Development (ENEA), Casaccia Research Centre, Via Anguillarese 301, 00123 Rome, Italy; antonio.rinaldi@enea.it; 2 Department of Materials, Environmental Sciences and Urban Planning (SIMAU), Università Politecnica delle Marche, Via Brecce Bianche 12, 60131 Ancona, Italy; t.bellezze@univpm.it (T.B.); m.l.ruello@univpm.it (M.L.R.); 3 Institute of Mechanics, Montanuniversität Leoben, Franz-Josef-Strasse 18, 8700 Leoben, Austria; e.gamsjaeger@unileoben.ac.at; 4 Department of Metals and Corrosion Engineering, University of Chemistry and Technology, Prague, Technicka 5, 166 28 Prague 6, Czech Republic; panovak@vscht.cz; 5 Faculty of Technical Sciences, University of Novi Sad, Trg Dositeja Obradovica 6, 21000 Novi Sad, Serbia; sebab@uns.ac.rs; 6 National R&D Institute for Nonferrous and Rare Metals, Biruintei Blvd. No. 102, 077145 Pantelimon, Romania; rpiticescu@imnr.ro

**Keywords:** critical raw materials, substitution, alloys, composites, extreme conditions, cobalt, tungsten, chromium, niobium, magnesium

## Abstract

In Europe, many technologies with high socio-economic benefits face materials requirements that are often affected by demand-supply disruption. This paper offers an overview of critical raw materials in high value alloys and metal-matrix composites used in critical applications, such as energy, transportation and machinery manufacturing associated with extreme working conditions in terms of temperature, loading, friction, wear and corrosion. The goal is to provide perspectives about the reduction and/or substitution of selected critical raw materials: Co, W, Cr, Nb and Mg.

## 1. Introduction

**Figure 1:**
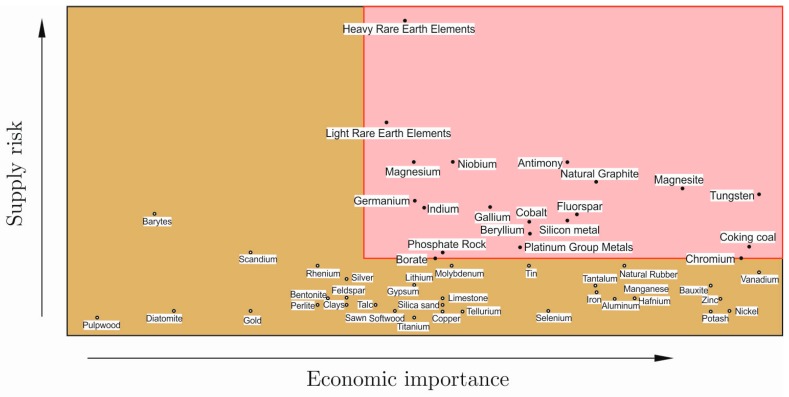
List of critical raw materials (CRMs) for the EU. Elements in the pink box are the 20 considered as most critical (reprinted from [2]).

Secure access to metals and minerals needed for sustained economic production is a growing challenge. Reduced access to raw materials may depress vital industrial sectors, in particular construction, automotive, aerospace and machinery manufacturing industries and chemical engineering, which may indeed be affected by a limited access to some critical raw materials (CRMs), for example in the EU. There is a clear unbalance in the European import/export flow of raw materials, featured by an exceeding amount of imports for all of them. To face the CRM problem, Europe indeed launched the “Raw Materials Initiative” to ensure access to raw materials [1]. In 2010, the Study on Critical Raw Materials at the EU level was carried out in order to identify the non-energy raw materials considered as critical for Europe: a list of 14 critical materials was produced on the basis of economic importance and the risk of supply disruption. The list was updated to the 20 most CRMs in 2014 (Figure 1) [2].

On 25 September 2013, the high-level steering group of the European Innovation Partnership (EIP) on Raw Materials released the Strategic Implementation Plan (SIP) [3], describing how to act for ensuring a sustainable supply of raw materials to the European economy and make Europe a world leader in raw materials exploration, extraction, processing, recycling and substitution by 2020. On 9 December 2014, the European Institute of Innovation and Technology (EIT) appointed the Knowledge Innovation Community (KIC) “EIT Raw Materials”, devoted to mitigating the risk of supply shortage of raw materials for the EU [4]. The above-mentioned KIC, a consortium composed of more than 100 partners all over Europe, has the role to “boost the competitiveness, growth and attractiveness of the European raw materials sector via radical innovation and entrepreneurship, integrating multiple disciplines, diversity and complementarity along the three sides of the knowledge triangle (business, education and research) and across the whole raw materials value chain” [4].

From a scientific and technological standpoint, different actions can be pursued to address the CRM issues, namely: (1) improving raw materials production processes (increase sustainable mining, reduce extraction costs, increase materials efficiency, increase safety, etc.); (2) finding suitable substitutes to partially or totally replace CRMs; (3) boosting recycling. 

According to the ERA-MIN (Network on the industrial handling of raw materials for European industries) Roadmap [5], “substitution means replacing critical elements in already existing materials by more abundant ones, using novel materials with no or reduced critical elements and increasing material efficiency in products and production”. “CRMs substitution is a complex process requiring visionary policy responses that are able to anticipate the different aspects of, and the far-reaching impacts on, research, industry, markets [6]”. The reliability of the substitute materials and the time needed for the “novel” products to be ready to enter the market are crucial points to consider, in order to avoid, at the laboratory scale, costly, but not scalable efforts. Moreover, new standardization procedures are required when critical elements are substituted or reduced in novel materials. It is a common opinion that substitution of CRMs should result in innovative materials with comparable or improved performances, easily and quickly integrable in the production processes, with improved potential for disassembling and recycling, lower risks for environment and human health and lower prices. In addition, reuse and recycling of scraps and end-of-life products for producing secondary raw materials for less demanding applications must be pursued to reduce the EU’s raw materials dependency and contribute to a better management of wastes. Economic efficiency, social equity and the “value chain design”, which is an essential methodology to identify barriers in the flow defined by the CRMs’ substitution and creation of a new product to be inserted into a particular industrial chain and market, should be also taken into account. 

Substitution of CRMs for application under extreme conditions is an urgent demand, because extreme conditions of temperature, wear and corrosion frequently occur in many applications: energy (concentrating solar power systems, thermoelectric devices, oil, gas and nuclear plants); transportation (automotive, marine, aeronautics, aerospace, etc.); manufacturing (cutting tools, high-speed machining, cryogenic machining, etc.). Some recently-appointed EU projects [7,8,9,10] identified selected critical raw materials used in the above-mentioned fields to be investigated for potential substitution: (1) Co and W, in WC-Co cemented carbide wear-resistant tool materials; (2) Cr and other CRMs in steels and coatings; (3) Nb in high-strength low-alloy (HSLA) steel; (4) Co and other CRMs in high-temperature Ni-based superalloys; (5) Mg in alloys and superalloys.

In this paper, the state of the art on the role of the above-listed CRMs is described, and some strategies for the reduction of their content or their complete substitution in selected applications are proposed. The focus will be on the substitution of critical elements in bulk materials, in particular Cr in steels due to the widespread use of these alloys, while only a few remarks will be made on CRM-based coatings. The recycling issue is beyond the scope of this review and has to be considered separately in future works.

## 2. State of the Art

### 2.1. Co and W in WC-Co Cemented Carbide Wear-Resistant Tool Materials

**Figure 2:**
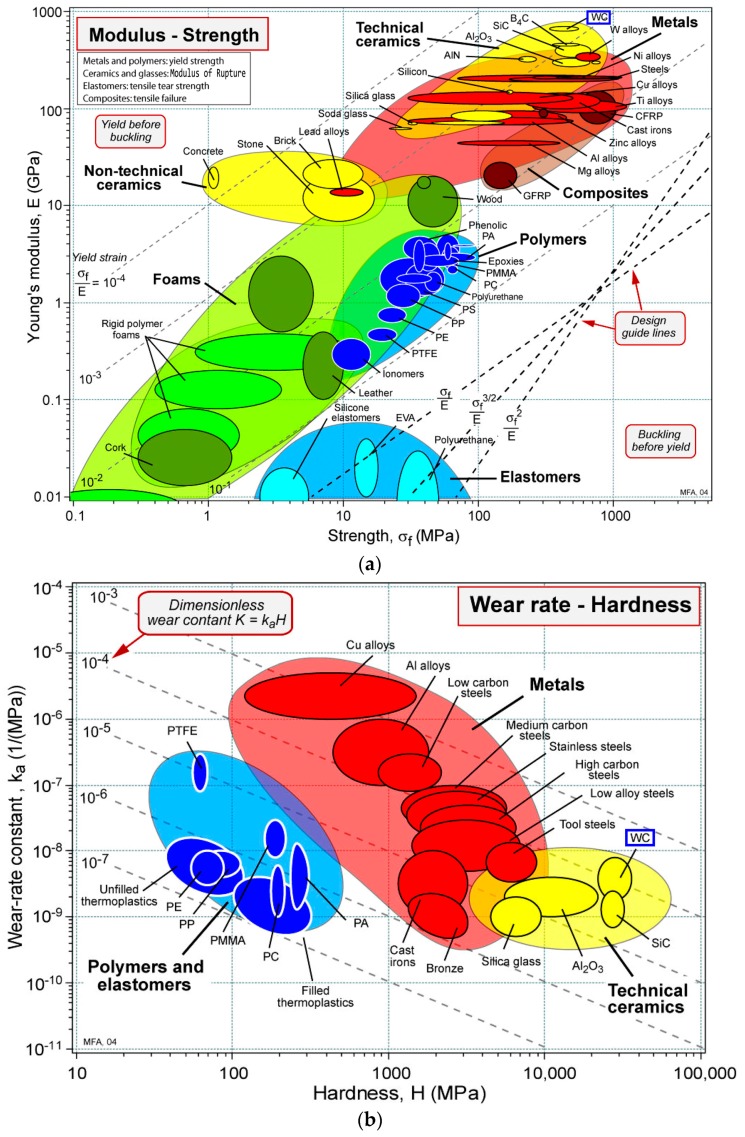
Ashby diagrams for different materials. (**a**) Young’s modulus vs. strength, (**b**) wear-rate constant vs. hardness. The blue rectangle highlights the WC cemented carbides (reprinted with the permission of Michael F. Ashby [13]).

The cemented carbides or hard metals are a range of composite materials that consist of hard carbide particles bonded together by a metallic binder. A wide variety of shapes and sizes can be produced using modern powder metallurgy. The proportion of carbide phase is normally 70%–97% of the total mass of the composite, and its grain size ranges between 0.2 μm and 20 μm [11]. Grades with binder content in the range of mass fraction of 3%–10% and grain sizes below 1 μm have generally the highest hardness and compressive strength values. The decrease of carbide grain sizes increases hardness, compressive and bending strength, but decreases impact strength and fracture toughness, while an increase in the binder phase produces lower hardness, lower Young’s modulus and compressive strength, but increases bending strength and fracture toughness. Cemented carbide consisting of tungsten carbide in a matrix of Co (WC-Co), where tungsten carbide (WC) is the hard phase and cobalt (Co) the binder one, is the basic cemented carbide structure, and its unique combination of strength, hardness and toughness satisfies the most demanding applications (see Figure 2) [12,13]. As pointed out in [14], the mechanical properties of cemented carbides mainly depend on the WC grain size, WC size distribution and on the Co content, where a higher content of Co leads to higher toughness and small carbide grains increase the hardness. Thereby, the excellent mechanical properties of this composite material can be achieved over a wide temperature range [15] and can be tailored for certain applications, e.g., as cutting tools for processing and shaping of metals, wood, graphite, composites and ceramics and also in wear-resistant parts and components in various industrial and mining applications. Cemented carbide is the preferred composite for components that must withstand wear and exhibits a high degree of toughness providing longer life in applications where other materials would fail prematurely [15,16]. Around 12% of the consumed Co is used in hard metals [17]. Co is the most widely-used metal binder in cemented carbides due its excellent wetting, adhesion and mechanical properties, while W is the metal with the highest melting point among all pure components and, due to its hardness, density and high corrosion resistance, finds the largest application (about 60%) as tungsten carbide WC in hard metals.

WC is usually produced by a controlled solid-state reaction between W and C. Since this process is expensive due to the required high temperatures and long heating time and it results in large WC grains, tungsten salts or tungsten oxides were recently proposed as W precursors and new production routes (e.g., microwave synthesis and mechanical alloying) have been suggested [18]. Substitution or optimization of Co and W in the tooling industry is a primary target of broad impact and great complexity. 

Within the tooling industry, coatings are also another important aspect. It is estimated that 80%–90% of hard metal tools sold today are coated to increase their wear resistance and long life performance [19]. Examples of coatings, some of which contain CRMs, include CrN, TiN, WC-Co, WC-Co-Cr, TiC, Ti(C)N, TiAlN, ZrN, ZrAlN, DLC (diamond-like carbon) and Al_2_O_3_. Coatings are deposited by different techniques: physical vapor deposition (PVD), chemical vapor deposition (CVD), plasma spray, electroplating, laser deposition, HVOF (high velocity oxy fuel) thermal spray, sol gel, etc.

### 2.2. Co and Other CRMs in High-Temperature Ni-Based Superalloys

Co and/or Ni-based superalloys represent jointly one of the largest Co markets. In Ni-based superalloys, Co is an alloying element with the role of stabilizing and solution strengthening the γ phase, together with other alloying elements (Cr, Mo, Fe and W). The essential solutes in Ni-based superalloys are Al and/or Ti (the total mole fraction is typically less than 10%) required to form the characteristic γ′ phase, an intermetallic compound according to the formula Ni_3_(Al,Ti). Strength usually decreases in metals with increasing temperature; however, strength in Ni-based superalloys is comparatively insensitive to temperature due to γ′-precipitates [20]. The amount of γ′ phase depends on composition and temperature, and it confers elevated-temperature strength and resistance to creep deformation to the material [20]. Additional strengthening at low temperatures can be achieved by the γ′′ phase with the composition Ni_3_Nb or Ni_3_V in case of additions of Nb (Inconel) or V, by solid solution strengthening of γ phase, and by oxide dispersion strengthening (ODS) [21]. ODS superalloys are produced by a mechanical alloying process and are particularly suitable for applications at elevated temperature. Ni-based superalloys are characterized by high melting points, high values of strength and corrosion resistance at elevated temperature and thermal fatigue resistance, together with a high weldability, which makes them suitable for applications as turbine discs and turbine blades in the turbine engines for aerospace, marine and land-based power generation applications. They remain the reference candidates for the hot-sections of next generation power plants (both conventional and concentrated solar) with a higher temperature point of operation and may withstand loadings at temperatures close to their melting point, a mix of properties that makes their replacement a hard task. At the same time, such properties are achieved by CRM alloying elements, such as Cr, Co, Y, W, Nb, etc. Tian et al. [22] studied the effect of Co on the mechanical properties of Ni-based superalloys. They observed that Co enhances the yield strength and the strain hardening capacity, but a higher Co content sharply decreases the ductility at higher temperatures. Effects of composition on the oxidation resistance of Ni-based superalloys have been investigated by Park et al. [23]. Recently, creep strength and oxidation resistance of Co-Ni-Al-W-Cr model superalloys from pure Ni-based to pure Co-based superalloys have been studied by Zenk et al. [24]. They found that high Ni contents decelerate the oxidation kinetics. According to [24], the creep strength of Ni-based alloys reaches a maximum at a certain Co content, but the creep properties deteriorate dramatically at higher Co additions. Cr and Al are well known to improve the oxidation resistance because they form Cr_2_O_3_ or Al_2_O_3_, respectively, acting as protective barriers. Cr is the most important element in Ni-based alloys to prevent high-temperature oxidation and corrosion. On the other hand, at very high Cr content (30%–40%), however, the precipitation of a Cr-rich phase may reduce the mechanical properties and processability of the alloys [25]. 

In the fourth generation Ni superalloys, a mass fraction of 2%–3% Ru (a platinum group metal element, critical as well) is added to hinder the precipitation of topologically-close-packed (TCP) phases [26,27] and improve the high-temperature microstructure stability. It is shown that the nucleation rate of TCP is reduced due to Ru [28]. In fourth generation superalloys, the creep strength increases, so that these alloys have capabilities to stand higher temperatures with respect to the previous generation superalloys. In fifth generation superalloys, the increase of Ru content (up to 5%–6%) together with the accurate control of γ and γ′ phases, allows one to further increase creep strength [26]. However, commercialization of fourth and fifth generation Ni-based superalloys is prevented by their reduced resistance against oxidation (due to the increased content of refractory metals, such as Ru, Mo and Re); therefore, sixth generation Ni-based superalloys are under investigation to increase creep resistance without decreasing heat-oxidation resistance. The content of Co in the proposed alloys varies between 9.5% and 16.5% [26].

### 2.3. Cr in Alloys and Surface Coatings

There are more than one hundred Cr-containing metallic alloys, each one developed for a particular need and with particular properties. The most important characteristics common to all Cr-containing alloys, including stainless steels, is that they contain a sufficient amount of this element that strongly increases corrosion resistance, oxidation resistance and/or heat resistance.

Stainless steels and, in general, all stainless alloys represent fundamental metallic materials for industrial and civil applications within the EU and they are a pillar for its economy. The EU produces 21.1% of worldwide steel output [2], second only to China (45.5%), but does not have easy access to the raw materials (i.e., Ni, Cr, Mo, etc.) required for producing these alloys. The supplying of these elements from other countries is an issue related to their restriction on exporting raw materials in the international market. The reasons for the restrictions in the accessibility of raw materials for, e.g., the production of stainless alloys often results from an uncertain political stability of such countries and/or in their exporting strategies, based on their own limited manufacturing capacity. Civil wars in Africa and Asia and the hopefully-resolved Cold War between powers in the Western and the Eastern-Bloc countries may serve as examples for such political instabilities affecting the access to raw materials for the EU [29]. 

As an alloying element in stainless steels, Cr ranges between a mass fraction of 10.5% and 30% [29,30]. Owing to its strong reactivity with oxygen, it provides the ability to passivate the surface by an adherent, insoluble, ultrathin (1–2 nm) film that protects the underlying metal against attacks of the corrosive agents, mainly in acids and/or chloride-containing environments. For a stable passivation, the mass fraction of Cr should be at least 10.5% [29,31,32,33]. The presence of Cr in the alloy not only guarantees the protection against corrosion, but Cr is also responsible for self-healing in the presence of oxygen (e.g., mechanical scratches or surface damage are coated by an oxide layer and thereby regain their corrosion resistance) [29,32]. Thanks to the formation of continuous Cr_2_O_3_ scale on the alloys surface, Cr gives to these metallic materials the necessary protection in the high-temperature applications [29].

Cr falls in the “criticality zone” in Figure 1 and represents a metal of a high economic and strategic relevance. “The recycling rate of Cr is low and there are limited options for substitution, particularly in its main application, i.e., in stainless steel” [2].

In Section 3.3, a critical review of a possible partial substitution of Cr in stainless steel alloys will be proposed, even if its substitution will remain a utopia (or “a dream” [31]) in applications, such as severe aqueous environments, where high corrosion resistance steels are required. In the typically used alloys of the 300-series, as AISI 304 and AISI 316, the mass fraction of Cr reaches 18%, representing therefore a suitable amount for guaranteeing a corrosion resistance in many moderately severe environments. These well-established alloys can be considered as standards and new alloys with reduced Cr content have to perform in a similar way. Apart from its application in bulk alloys, Cr is largely used as conversion chromate coatings, hard chrome and PVD CrN, to improve the resistance to high temperature, corrosion and wear of coated products. In recent years, a great effort has been devoted to looking for a replacement for Cr plating, driven also by its health risk. Electroplated Cr solutions and conversion chromate coatings contain in fact hexavalent Cr, which has recognized cancerogenic effects and must be banned in a short time period according to recent EU directives (2002/95/CE 27-01-2003). Cr (III)-based conversion treatments are extensively studied, because they are considered to be commercially-acceptable and environmental-friendly alternatives to conventional Cr (VI) passivation treatments for several applications [34,35].

Although Cr (III) eliminates the health risk of Cr (VI), it does not solve Cr criticality, and therefore, alternative coatings are needed.

### 2.4. Nb in Stainless Steel and High-Strength Low-Alloy Steels

Nb is used to improve the corrosion resistance and high-temperature resistance of the steel. Its use worldwide for the different grades of steel is about 3%.

HSLA steels are a work horse for mass production applications, such as automotive, bridges, cranes, etc., where high strength and lightweight are required at relatively low costs. HSLA steels are also needed in the case of severe conditions of pressure and temperature (e.g., during welding) and are expected to transport fluid at high flow rates (e.g., in gas and oil pipelines). HSLA steels contain several alloying elements that modify the microstructure of carbon steels through precipitation strengthening and grain refinement. The role of Nb in HSLA is to provide dispersion strengthening by forming NbC or Nb(C,N) (in the case of N-containing steels). Nb significantly retards static recrystallization and grain growth in hot-rolled austenite, enabling rolling under non-recrystallization conditions [36]. Microalloyed HSLA steels have been developed for the automotive industry with the aim of reducing weight without losing strength. Advanced high-strength steels (AHSS) for automotive applications continue to evolve and new commercialized AHSS that exhibit high strength and enhanced formability required for manufacturing are being offered around the world. By means of these steels, cost and weight can be effectively reduced with an even improved performance [37].

### 2.5. Mg in Aerospace Industry Al-Alloys

Magnesium is the lightest metal element and its primary use is in structural alloys. One of the most significant application of Mg in engineering is as an alloying element in aluminum alloys used in aircraft and military industry in general. Although the amount of Mg in AA7000 series alloys is relatively small and amounts to a mass fraction of around 2%–5% (mole fraction up to 5.5%), the annual demand is large [38,39]. These alloys are used for the fabrication of aircraft skins (especially upper wing), stringers, landing gears, as well as horizontal and vertical stabilizers. The most significant property of these alloys is their relatively high strength compared to other aluminum alloys, more specifically tensile and compression strength. Furthermore, there are other advantages, such as high strength-to-weight ratio, ease of machining and relatively low cost. On the other hand, these alloys are susceptible to corrosion. Combined with fatigue, the corrosion can shorten the life of the aircraft structures, increasing the demand for maintenance and cost in general. Mg in these alloys plays a major role, enabling age hardening through the precipitation strengthening mechanism of spherical coherent GP (Guinier-Preston) zones rich in Mg and Zn [40]. The most significant 7000 alloys are: 7050, 7055, 7075, 7150 and 7475 [39].

## 3. Possible CRM-Friendly Solutions

### 3.1. Alternatives to W and Co in WC-Co Cemented Carbides

Both Co and WC may be partially or totally replaced in WC-Co cemented carbides, even if the known substitutes may result in lower mechanical strength. Substitution of WC cemented carbide seems possible in most of the applications apart from machining at very extreme loads [16]. Research in this field is very active, and most of the competing composites are produced at lower costs, even if with some loss in performances [17,41,42]. WC cemented carbides may be substituted by tool steels, ceramics and cermets in various applications [43,44]. Cermets combine the advantages of ceramic and metals and offer the advantages of being lightweight if compared to WC-based hard metal [45]. W-free carbides and cermets based on Ti interstitial phases, such as TiC or TiCN in the metallic binder phase of Ni, are commercially made in Europe, even though their fracture toughness and high-temperature properties are actually inferior to those of WC-Co materials. Furthermore, in Japan, where national regulations indicated the need to reduce the use of some CRMs (W, In and Dy), TiCN-based cermets with a Ni binder are used as WC-Co alternatives in some applications [46]. As another example, a 20% reduction of W was proposed by Ishida et al. [47], who found a combination of WC with the cermet TiCN-Co/Ni, having the same level of fracture resistance and wear resistance as the WC-Co cemented carbide. Partial reduction of W in WC may be also pursued by employing composite ceramic materials based on Al_2_O_3_ or MAX phases [48,49,50]. These latter are a family of layered ternary carbides or nitrides with the general formula M*_n_*_+1_AX*_n_* (MAX) with *n* = 1–3, where M is an early transition metal, A is an A-group element and X stands for C or N. Apart from their ceramic properties, these materials exhibit also some metallic characteristics, such as machinability, toughness and electrical conductivity.

The Co substitution in WC-Co is also driven by the fact that Co has recognized genotoxic and cancerogenic activity, with even higher toxicity effects if combined with WC [12]. Very recently, Tarrago et al. [51,52] investigated new binder formulations (in collaboration with SANDVIK Hyperion), featuring total or partial replacement of Co with Ni and other metals, and found experimentally that hardness decreased and fracture toughness increased as binder composition was shifted from Co to Co-Ni. However, flexural strength did not depend on the composition of the binder. Furthermore, binder composition for similar microstructures (i.e., similar carbide size and volume fraction of binder) had negligible influence on the fatigue crack growth (FCG) threshold and the fatigue sensitivity. By a CRM substitution standpoint, these results are significant, as they may suggest that Co can be replaced or partially substituted with Ni at least in some hard metal applications. As other alternative binder metals, alloys such as Ni-Fe, Ni-Fe-Co or intermetallic compounds such as Ni_3_Al, NiAl, Fe_3_Al and FeAl are investigated owing to their good wetting behavior. In Ni-Fe and Ni-Fe-Co binders, the careful control of the Fe/Ni ratio, of carbon content and of heat treatments allows the desired degree of metastability of the binder phase to be achieved, leading to increased toughness with respect to the conventional WC-Co, without losing significant hardness [12,53,54]. The composites having NiAl matrix and ceramic reinforcement (Al_2_O_3_) were developed and successfully tested. However, the wear resistance does not reach the performances of WC-Co, but it exceeds the properties of current cold work tool steels without the need for heat treatment [55]. Current research results also indicate that the ternary Fe-Al-Si and Ti-Al-Si alloys exhibit excellent wear resistance [56]. However, these materials, similarly as many other materials based on intermetallics, are relatively brittle at room temperature. These problems are currently being solved by modification of the chemical composition using other alloying elements [57] or modifying the production technology. The modern powder metallurgy processes able to produce nanostructured materials seem to be promising solutions, which could improve the ductility of these alloys and bring them even to the application as tool materials [58].

In addition, the application of CRMs-free coatings, such as TiAlN, may be used to improve the properties of other types of tool materials to reach performances comparable with those of WC-Co tools [59].

### 3.2. Alternative to Co and Other CRMs in High-Temperature Ni-Based Superalloys

Superalloys are commonly employed in last generation supercritical steam turbines operating at high temperature. Ti-based alloys are used when being lightweight is requested, but their applications are limited to temperatures below 700 °C due to their poor oxidation resistance. 

It seems to be a challenging task to find the minimum Co content required to achieve the optimum tensile properties or to substitute Co by a less critical element, which also enhances yield strength and strain hardening capacity. In addition, it is difficult to reduce Cr due to its fundamental role in providing corrosion resistance of these alloys.

Ni-based ODS superalloys are, however, at present, a competitive “substitutive” option to Inconel 718 and Alloy 800 in many applications. ODS superalloys are produced starting from alloy powders and yttrium oxide, using the mechanical alloying process. Yttria (mass fraction of about 1%) is finely dispersed in the alloys and, due to the fact that it is a very stable oxide, it makes the alloy particularly suitable for elevated temperature applications. Ni-based ODS superalloys such as the PM1000 have excellent high-temperature creep-rupture strength and excellent oxidation resistance [60]. At present, ODS limited use in aircraft turbines is due to the manufacturing complexity. However, these alloys still contain a reduced amount of Cr and, in addition, Y as CRMs. Y can be further minimized by using Al in solid solution. In several applications, Ni-based superalloys are successfully substituted by using intermetallic TiAl materials. The most important use of these materials in high-temperature applications is in jet engines produced by GE Aviation [61]. The role of the microstructural design of a cast and heat-treated intermetallic multi-phase γ-TiAl-based alloy was demonstrated in [62]. These γ-TiAl-based alloys are promising candidates for lightweight high-temperature applications. In the chemical industry, the Ni superalloys can also be substituted by iron aluminides (FeAl or Fe_3_Al), which have excellent resistance to oxidation and sulfidation at high temperatures [63,64].

Recently, Co-Ni-based superalloys with a near-zero γ-γ′ lattice misfit have been investigated by atom probe tomography [65]. It has been observed that the partitioning ratio of W is reduced for superalloys with high Co/Ni ratios, i.e., the solid solution strengthening component of the γ phase is increased. The partitioning behavior of the solutes in advanced Co-Ni-based alloys may result in improved mechanical properties for a certain composition. 

A promising alternative to superalloys is constituted by ceramic matrix composites (CMCs) thanks to their strength and toughness. In 2010, GE reported for the first time the development of CMCs-based turbine blades. Substitution of superalloys in a gas turbine by CMCs would provide an increased efficiency due to weight reduction [17,66]. 

**Figure 3:**
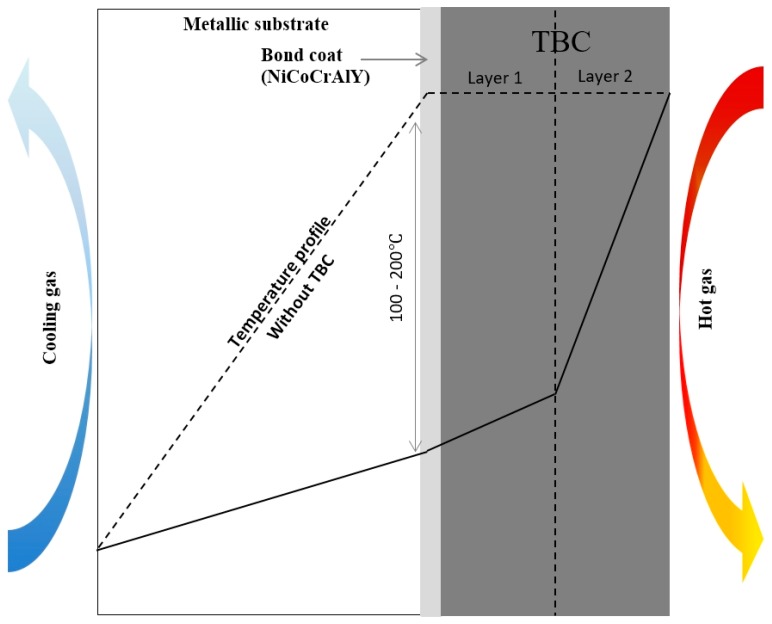
Schematic representation of ceramic thermal barrier coatings. TBC, thermal barrier coating.

Thermal barrier coatings (TBCs) are another alternative to reduce the CRMs in high-temperature applications, such as turbine parts in energy and aeronautics applications. The role of TBC is to decrease drastically the operating temperature of the metallic part in order to preserve their mechanical properties (Figure 3). Alumina, mullite, yttria-doped zirconia, ceria or perovskites such as barium zirconate and zirconium lanthanide are generally used for such applications [67] due to their high melting point and low thermal conductivity. It is also essential for the required properties that these materials are not subjected to transformations between room temperature and operation temperature.

**Figure 4:**
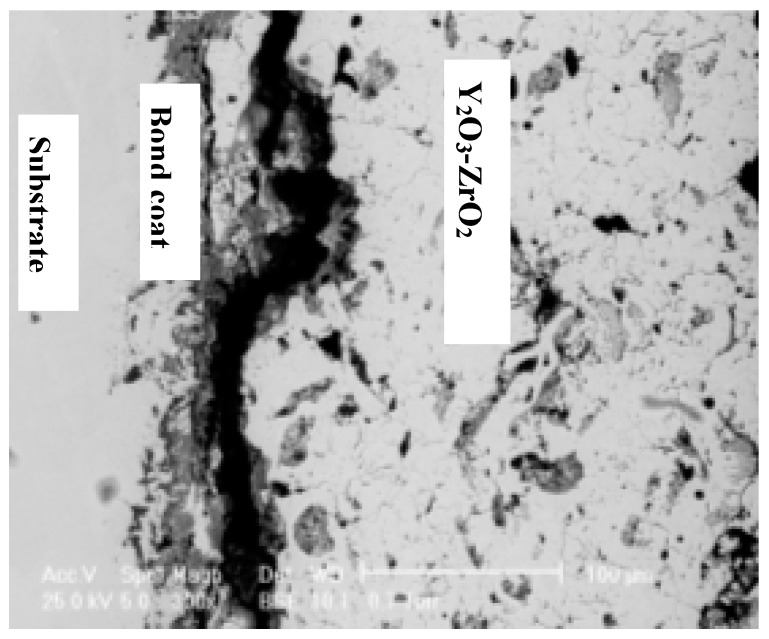
SEM image of the NiCoCrAlY bonding coat/Y_2_O_3_-ZrO_2_ coating (reprinted from [69]).

With respect to manufacturing TBCs, air plasma spraying and electron-beam physical vapor deposition are the best available industrial coating technologies. The use of high-temperature Ni- and Co-based superalloys is reduced to the thin (usually 1 μm) bonding coat layer. Their successful application depends also on the good adherence to the metallic substrate [68]. The formation of closed micro-pores inside the ceramic nanostructured coating (Figure 4) enhances the thermal shock resistance of the coating [69].

### 3.3. Alternative to Cr in Stainless Alloys and Surface Coatings

Taking into account that Cr cannot be totally eliminated in stainless alloys, because it is absolutely necessary for increasing their corrosion resistance, it is possible to reduce its amount by substituting it partially with other elements (as for example, Al, Si, etc.) in solid solution.

Many U.S. researchers worked on the partial substitution of Cr in stainless steels during the 1980s and 1990s, because it was considered important due to the possible import vulnerability of the U.S. in supplying strategic materials [70].

First of all, a distinction between the resistance to oxidation in high-temperature applications and the resistance to aggressive electrolytic solutions must be done, when searching for alternative elements to Cr in stainless steel alloys while improving or at least maintaining their corrosion properties. In any case, only a reduction of Cr content is possible for obtaining new stainless steels with comparable corrosion properties to those of typically used 300-series, passing from 18% of these alloys to 8%–12% [31,70,71], reducing in this way one-third the consumption of Cr. Actually, this is possible in less severe exposure conditions, but in practice, with particularly demanding ones, this represents a huge challenge: a further reduction of Cr and a complete elimination of this element from stainless steel alloys is considered unfeasible [70,71].

**Figure 5:**
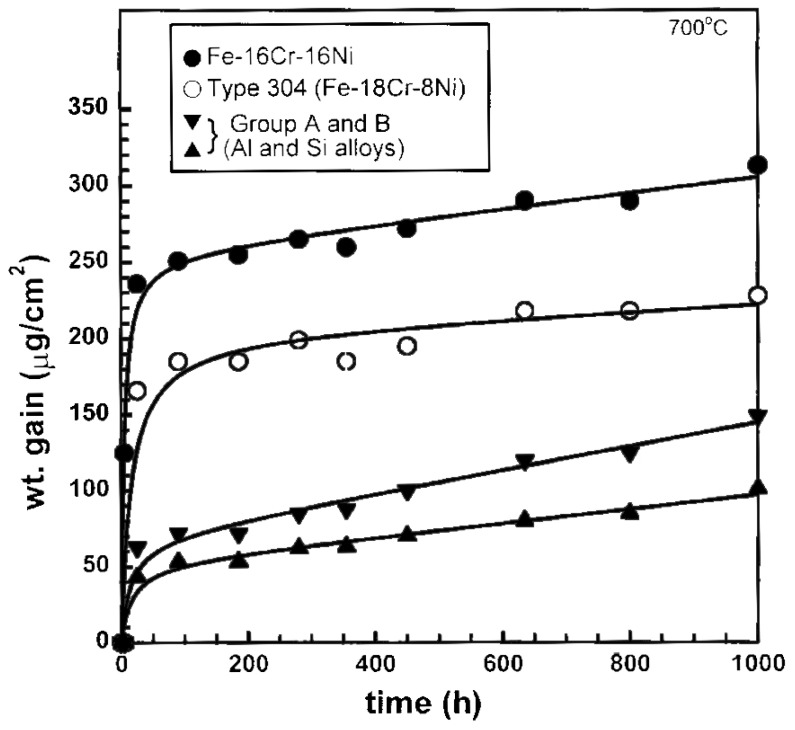
Oxidation behavior of Fe-16Cr-16Ni alloy in comparison with Type 304 alloy, alloys with Si + Al additions (Group A) and alloys with only Si additions (Group B) (adapted from [73] with permission of Springer).

Possible elements for substituting Cr are Si and Al in applications where corrosion at high temperatures occurs [71]: (8-10)Cr-(10-14)Ni-(0-8.5)Si-(0-4)Al alloys were studied in comparison with AISI 304 and AISI 410 (a ferritic 12% low-chromium steel). Good results have been obtained when 8% or 10% of Cr and 5% of Si were used in exposure tests carried out at 700 and 800 °C, not only in terms of corrosion resistance, but also in terms of mechanical properties. Other findings in this direction [72,73,74] reported the efficiency of Si and Al in enhancing the high-temperature oxidation properties of stainless steel and in general of Fe-Cr-Ni alloys, with particular emphasis on Si action [73], and significant improvements obtained with Al additions at 700 °C (Figure 5).

Instead, at 800 °C, the presence of Al impairs the oxidation resistance properties of the new examined alloys [73]. This has been explained by considering the higher affinity of Al for oxygen with respect to Si, and therefore, at high temperatures, the preferential formation of aluminum oxide limits the formation of the more protective SiO_2_ film, present in the Si-only alloys [73]. On the other hand, in Fe-Cr alloys, where Cr content reaches a percentage of 40% [75], a partial substitution of this element by Al is considered effective in high-temperature applications (up to 1375 °C, in preoxidized conditions), thanks to the formation of a stable and protective α-Al_2_O_3_ layer. In addition to Al, microalloying with Ru (0.2%) further enhances the corrosion properties of Fe-Cr-Al alloys thanks to the formation of Cr-rich layers between the aluminum oxide layer and the substrate. Such a high amount of Cr, in any case, guarantees the durability of the metallic material (20% of Cr was found not sufficient to limit the oxidation at high temperature). 

**Figure 6:**
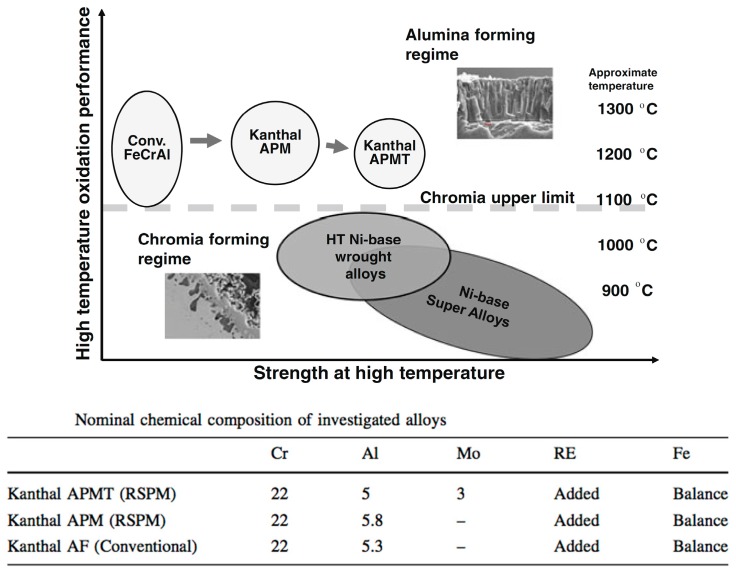
Fe-Cr-Al oxide dispersion strengthening (ODS) (KANTHAL©) currently produced or previously investigated at SANDVIK for very high temperature, 900–1350 °C (credit SANDVIK). The comparison from sagging test between a KANTHAL© ODS and a Fe35Ni25Cr superalloy after 2300 h at 1100 °C in carburizing atmosphere shows greater damage (oxidation and creep) rate in the superalloy. RSPM stands for rapid solidification powder, RE stands for rare earths and HT stands for high temperature (reprinted from [76], with permission of Bo Jönsson and with permission of Springer).

From a commercial standpoint, for over a decade, CARPENTER in the USA and SANDVIK in the EU have been actively researching alumina-forming steels, both austenitic and ferritic, with partial substitution of Cr by Al. As an example, cast ferritic Fe-Cr-Al steels could represent a way to reduce Cr by over 30% compared to stainless (austenitic) steels, with a penalty in terms of corrosion properties that may be acceptable in certain applications. Since standard alloys, such as AISI 316L, 347, 321, typically contain 18-20Cr, 10-14Ni and 2-3Mo, a CRM-optimized Fe-Cr-Al steel could have 10Cr-4Al, which would ensure a competitive price and a substantial Cr reduction. These systems are developed also in ODS forms by SANDVIK, being branded as KANTHAL© and having a higher content of Cr (22%) to be optimal for very high temperatures up to 1300 °C, thus directly competing with Ni superalloys. Properties are tunable and vary for different grades, as portrayed in the comparison in Figure 6, demonstrating a remarkable degree of flexibility of alumina-forming alloys. 

Other elements were proposed for partial replacement of Cr, such as Mo, Cu, V, N and Ni [31,70]; some of these elements are stronger ferrite stabilizers than Cr; therefore Ni, which is an austenite stabilizer, must reach a percentage up to 24%, with the consequence of increased material costs [31] and stainless steel price fluctuations [77] linked to the Ni price. 

Finally, in the field of oxidation at high temperatures, some attempts were done for the complete substitution of Cr by examining Fe-Al-Mo and Fe-Mn-Al alloys without reaching optimal corrosion performances if compared to those of 300 series alloys [70,78]. The austenitic Fe-31Mn-7.5Al-1.3Si-0.9C, used to obtain steel wires for high-temperature applications, was tested and it gave similar results as AISI 304 for temperatures up to 700 °C, but, the properties were inferior compared to AISI 304 at 800 °C [79].

For the corrosion performances of stainless alloys in electrolytic solutions, the substitution of Cr, even partially, is possible, but only in moderately corrosive environments [31,70]. Corrosion resistance is enhanced with an increasing amount of Cr in stainless alloys both in acidic environments and in those environments with a high concentration of chlorides [31,80]. From this basis, the reduction of this fundamental element in stainless alloys represents a huge challenge within the CRMs substitution and/or reduction under extreme corrosion conditions. 

It is concluded in a very old paper that the minimum amount of Cr that can be tolerated to find comparable performances to AISI 304 is equal to 12% [81]. With such an amount of Cr, an addition of 2%–3% of Si or Mo reduced the critical passivation current in the active corrosion of new alloys tested in H_2_SO_4_ 1N. The same composition showed an increase of pitting potential in NaCl 1N, while Al gave a decrease of this potential. Thus, it is recommended not to use in these alloys more than 1% Al or to eliminate it for applications in aggressive aqueous solutions [72]. 

Other authors [82] confirmed the beneficial effect of Si additions to steels with Cr ranging between 8% and 13%, in H_2_SO_4_ solutions (0.1–1 N) containing various amounts of sulfates, when C_Si_ + C_Cr_ was above a mole fraction of 14%. When this sum was above a mole fraction of 15%, beneficial effects on pitting potential were found in solutions with 0.2 g/dm^3^ of NaCl at neutral pH. The reasons for the alloy protection determined by silicon additions are not completely known; however, some hypotheses assign this effect to the stabilization of the protective film due to the formation of fayalite (Fe_2_SiO_4_). 

Si, Al and Mo are all ferrite stabilizers [81] and, to obtain an austenitic phase, which gives good mechanical properties to these alloys, elements like Mn and Ni should be taken into account. They stabilize the austenitic phase, but the corrosion performances are moderately improved by the addition of these elements; however, Ni (10%) is preferred in place of Mn, with the consequence of cost increase. In conclusion, the addition of 2% of Mo provides the best composition (12Cr-10Ni-1.5Si-1Al-2Mo) with a corrosion performance comparable to AISI 304 in moderately-acidic solutions and chloride-containing solutions.

**Figure 7:**
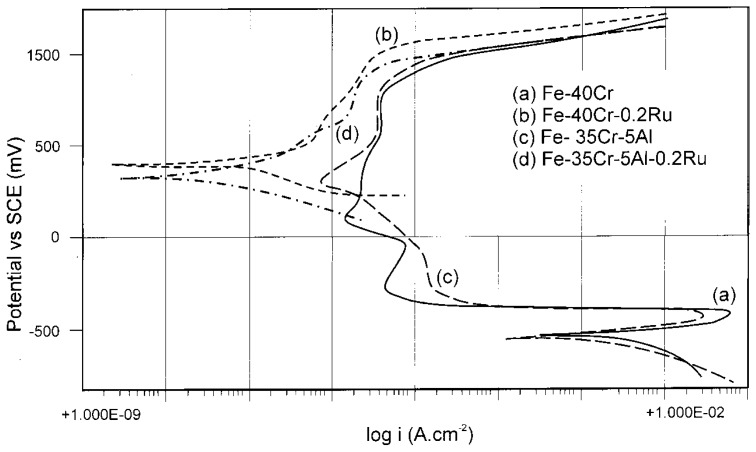
Potentiodynamic scans in 10% H_2_SO_4_ at 25 °C of Fe-40Cr alloy in comparison to an alloy where Cr is partially substituted by Al (Fe-35Cr-5Al) and the other two alloys where both are modified by micro-addition of Ru (0.2%) (reprinted from [75] with permission of Elsevier).

The scarce effect of Al in the partial substitution of Cr was found even in the above-mentioned high-Cr alloys (Fe-40Cr) [75]. In fact, the Fe-35Cr-5Al alloy gave a similar anodic behavior in a deaerated 10% H_2_SO_4_ solution at 25 °C with respect to the original alloy, while in a solution of NaCl 3.5%, there was a significant worsening in pitting corrosion resistance of the Al containing alloy. In these cases, a micro-addition of Ru (0.2%) not only improved high-temperature corrosion resistance of these alloys, but also the corrosion resistance in the above-mentioned aggressive solutions. Furthermore, self-passivation produced by the additions of noble metals like platinum-group metals (PGMs, CRMs, as well) occurs in alloys in contact with acidic solutions: these elements, by increasing the exchange current density of hydrogen evolution reaction, can bring the alloy towards passivation conditions. This phenomenon was observed in these alloys with Ru addition (Figure 7): in 10% H_2_SO_4_, the alloys showed a clear transition from active to passive behavior and, in more detail, it was observed that the amount of Cr can be reduced increasing the percentage of noble elements. In 3.5% NaCl solution, Ru additions produced an increase of pitting potential (about 200 mV) in Al containing alloy, even if it is always subjected to crevice corrosion.

Two alloys, Fe-8Cr-16Ni-Si-Cu with and without 1% of Mo, were tested in different acidic solutions and compared to the performance of AISI 304 [72]. Considering the very low amount of Cr in these alloys, Si and Cu were added to have the necessary corrosion resistance in aqueous solutions, but given their tendency to ferrite stabilization, Ni was brought from 10% of 304 to 16% to maintain the austenitic structure of the metallic material. Addition of Mo could be useful for corrosion resistance, while for high temperature oxidation, it is detrimental. Results showed that these alloys could be used in substitution of 304 stainless steels thanks to their comparable corrosion resistance performances.

Low Cr (15%) and low Ni (2%) Fe-Cr-Mn-Al-Ni alloys were also studied as alternatives to Fe-Cr-Ni alloys to obtain the well-known duplex stainless steels, adding therefore Al (1%–3%), which stabilizes the ferritic phase, and Mn (8%–12%), which stabilizes the austenitic phase [83]. Such alloys showed good pitting corrosion resistance in NaCl 3.5% at 25 °C and the localized phenomenon was observed in the austenitic phase due to the presence of Mn, which forms manganese sulfide inclusions responsible for the corrosion initiation.

Attempts to eliminate both Cr and Ni and to use Fe-Al-Mn alloys in place of Fe-Cr-Ni alloys in contact with aqueous solutions were done, but without success [84].

From the analysis made so far, the chances to reduce the use of Cr in stainless steels to obtain metallic materials with comparable corrosion performances of traditional alloys seems very low, mainly under extreme conditions. The partial substitution of Cr with Al is probably the most promising one, but only in applications where oxidation at high temperature occurs. At low temperatures, in the exposure applications to electrolytic solutions, the use of Si and PGMs is very interesting, but unfortunately, all of these elements are present in the CRMs’ critical area (Figure 1). However, the use of Ru in a very small amount (as micro-alloying element) can be taken into account, because it can bring beneficial effects in the self-passivation properties of low-Cr stainless alloys.

**Figure 8:**
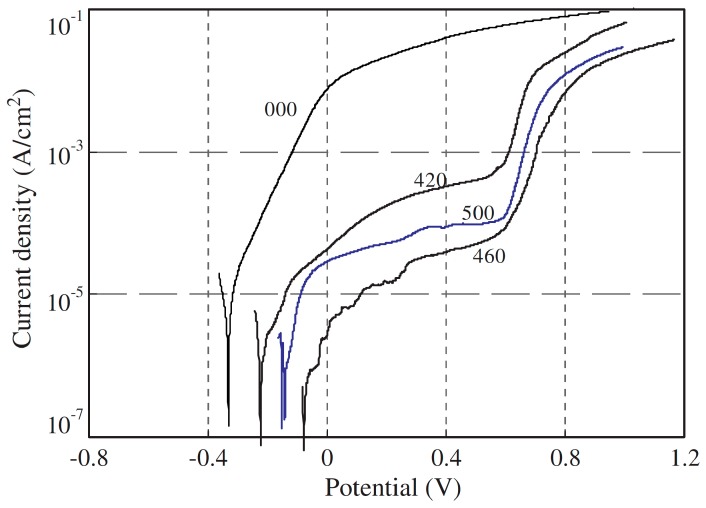
Polarization curves of untreated (000) and plasma nitrided (420, 500 and 460, where the number indicates the temperature of the nitriding process) 410 stainless steel in 3.5 wt % NaCl water solution (un-deaerated, unstirred) (adapted from [85] with permission of Elsevier).

Since Cr has the main function to prevent corrosion in stainless alloys at their surface, some strategies for saving the consumption of this element were practiced by using alloys with low-Cr content in the bulk, but producing a Cr-rich coating on the surface [70]. The methods used to achieve this result are based on laser technologies to obtain a surface alloying of Cr or ion implantation not only using Cr, but even C, N, etc. For example, an AISI 410 martensitic stainless steel with low-Cr content (12.2% Cr) has been plasma nitrided [85] at temperatures under 500 °C to avoid the formation of CrN and to promote the specific formation of iron nitrides. A material with significantly increased corrosion properties both in HCl and NaCl (Figure 8) solutions is obtained by this strategy. Other attempts for improving corrosion characteristics of stainless steels are to submit these materials to a passivation process [29,30,32,86] by using alloys with a Cr content under 18%. However, although the Cr content is not reduced by passivation of the alloys, their corrosion properties are enhanced [86]. Thus, this approach represents a good strategy for increasing the durability of such materials and then to save Cr.

As an alternative, a much reduced amount of Cr is used in ODS steels. New types of ODS alloys with controlled microstructure are able, in fact, to reduce the thermal stress and eliminate the use of alloys with high Cr content [87]. From a CRM substitution standpoint, they however contain a low amount of Yttria, and therefore, alternative Y-free ODS should be pursued. The substitution or minimization of Y may be obtained, as said before, by using Al in solid solution. 

Additive manufacturing is another major front where advances in substitution strategy and processing time are expected. Sustained trends in producing complex 3D-printed parts for energy applications are observed throughout industry, with pre-commercial achievements in printing high strength austenitic steels for turbines. Because the feedstock materials for the metal printing are often produced by gas-atomized powder technology, which is a mainstream technology to produce ODS, as well, the substitution opportunities discussed about ODS readily translate into an attractive extra benefit of next-generation additive manufacturing of steels, adding to the typical advantage of a reduced waste compared to traditional (subtractive) manufacturing.

Finally, hard chrome, PVD CrN [88,89,90,91,92] and Cr conversion coatings are very often used to improve the resistance to high temperature, corrosion and wear of components. Many types of CRM-free coatings based on single-layer or multilayer systems [93,94,95,96,97] or functionally-graded materials represent solutions to drastically reduce the content of Cr and other CRMs, especially for high temperature energy generation [98,99]. Moreover, non-chromate conversion coatings based on nanostructured Zr and/or Ti layers have the advantages of being free of toxic metals or organic compounds and are potential candidates to substitute chromate coatings [100].

### 3.4. Alternative to Nb in Stainless Steel and HSLA Steels

Ti, Ta and Mo are potential candidates to substitute Nb in stainless steel, even if sometimes with a cost increase [17]. In addition, high nitrogen (mass fraction of 0.5%) stainless steel can be used instead of Nb-steel in many applications due to enhanced hardness, yield strength, tensile strength, wear resistance and fatigue resistance. Similar to Ni, N in stainless steels stabilizes the austenite phase at room temperature. Additionally, it can increase the strength of stainless steels by accommodating at interstitial sites of the austenite solid lattice. 

In HSLA, Nb substitution seems more difficult. However, the amount of Nb required is small in HSLA with microalloyed Nb. Nb is required to obtain a fine microstructure. Thus, substitution of Nb in HSLA steels for oil pipelines seems rather unfeasible due to the superior toughness that Nb confers to these components and which is necessary to avoid fracture during extreme operative conditions (high pressures and high body forces (weight) over long distances) [17].

A more promising approach than substituting Nb can be pursued by further improving the properties of these CRM-containing steels. In situ measuring the evolution of the grain size and its distribution for different steel grades helps to obtain insight into the microstructural evolution during processing and service. It is on-going research [101,102] and remains certainly a challenging task to design new steels sustaining higher operating pressures with smaller wall thicknesses. In addition to modelling grain growth and thereby understanding the underlying processes, novel experimental techniques, such as high temperature laser scanning confocal microscopy (HT-LSCM) and laser ultrasonic technique (LUT), allow in situ monitoring of the microstructural evolution. Enhancing the lifetime of line pipes is in addition positive with respect to “health security and environment (HSE)” aspects by means of decreasing the frequency and the severity of possible problems.

### 3.5. Alternative to Mg in Aerospace Industry Al Alloys

**Figure 9:**
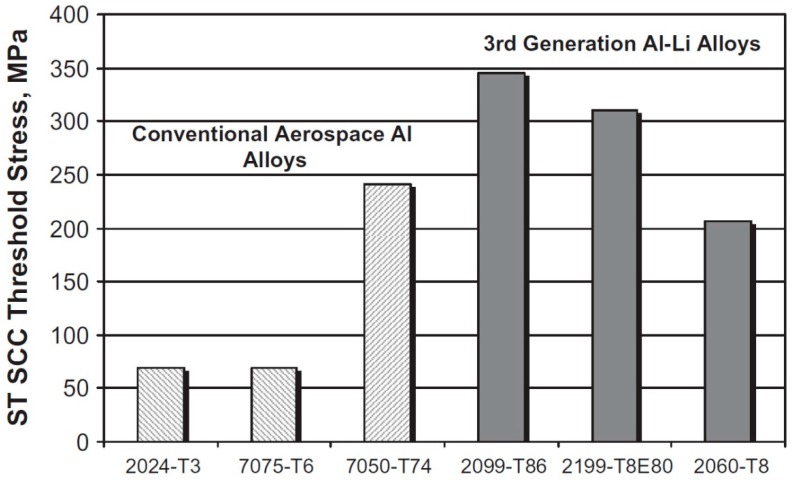
Comparison of corrosion resistance of Al-Li alloys with 2000 and 7000 series alloys. (reprinted with the permission of Tolga Dursun [39] and with permission of Elsevier).

An alternative to Al-Mg alloys is represented by the new generation of alloys containing Cu and Li. As Al-Mg-Zn alloys of the 7000 series were developed to partially substitute 2000 series of Al-Cu, new Al-Cu alloys with additions of Li are found as promising alternatives. These alloys are often referred to as third generation Al-Li alloys, although the Cu content is higher than that of Li and there is still a certain amount of Mg. However, the Mg amount is significantly lower: from 0.05% to 0.8%. Al-Cu-Li alloys are also age hardened, as Al-Mg-Zn alloys [103]. These Al-Cu-Li alloys, such as 2050-T84, possess an improved strength, toughness and corrosion resistance compared to 7050-T7451 and a 5% lower density, leading to a 10% weight reduction of components. Both 2050 and 2060-T8 alloys have a higher corrosion resistance than 7075-T6. Thus, 2050 and 2060-T8 alloys are a viable replacement for the fabrication of fuselage, lower wing and upper wing skins of aircraft [39,104,105] (Figure 9).

Another alternative to Al-alloys are in general composite materials reinforced with carbon fibers, mainly due to their superior specific strength and stiffness, as well as fatigue and corrosion resistance. However, their certification and fabrication costs are significantly higher, while their mechanical properties can be altered in different environmental conditions (cold/hot environments, moisture absorption). Fiber metal laminates that include Al-alloy layers and glass fiber-reinforced epoxy resin layers can be viewed as an alternative to carbon fiber composite materials. However, joining, repairing and recycling remain to be addressed [39,106,107]. Therefore, Al-alloys are still very important in aircraft industries, which can be further established by the development of new welding techniques, such as friction stir welding (FSW), which enables relatively high joint strengths, impossible to obtain with arc welding processes [108,109,110].

## 4. Outlook

It is evident that elucidating the role of CRM on the evolution of the microstructure during the processing and during service of high tech material is a precondition for future materials’ design aiming at the reduction or even substitution of CRMs. Success may depend in part on the identification of key niches where mainstream CRM-rich materials for extreme applications can be replaced either by CRM-friendly alternatives with more modest properties or be optimized in composition to boost performance and components’ life manifolds. 

Thermodynamically-based modeling in combination with experimental investigations represents a versatile tool to understand the governing processes occurring in materials. The development of modern steels, superalloys and other technologically important materials depends on a profound understanding of the function of alloying elements and in particular their role in the phase transformations. Examples for recent advances mainly in the field of steels are, e.g., reported in two review papers [111,112] from the ALEMI (Alloying Elements on Migrating Interfaces) group. By means of modern characterization techniques (in situ tension and bending experiments, high resolution backscatter diffraction mapping, transmission electron microscopy and synchrotron X-ray diffraction analysis) and thermodynamic modeling, it is demonstrated in [113] that due to a grain size-dependent competition between mechanical twinning and deformation-induced phase transformation, unexpectedly smaller austenite grains are less stable. It is well known that not only the composition, but also the thermo-mechanical processing controls the sophisticated microstructures and, thus, the final properties of functionally-oriented materials. The Rietveld method in combination with the double-Voigt peak broadening model was used to analyze microstructural changes from X-ray diffractograms of stainless steels [114]. As the microstructural parameters (e.g., dislocation densities, phase fractions) are related to the mechanical properties, appropriate heat treatments for engineering steels to obtain tailored mechanical properties can be suggested by this approach. Thereby, the mechanical properties of existing steels and other technologically important alloys could be improved or new alloys with a lower content of critical raw materials, but with the same performance could be introduced. In addition, additive manufacturing may lead to breakthroughs in substitution and time processing of high tech materials. Sustained trends in producing complex 3D parts for the energy industry are already observed throughout industry, with pre-commercial achievements in printing high strength alloys, such as Inconel 718 and austenitic steels. 

Finally, careful evaluation of sustainability, socio-economic and health impacts should be the driving forces of substitution approaches. Sustainable alternative materials should be targeted at reducing negative environmental impacts (CO_2_ emission, water waste, toxicity, etc.) and preserving natural capital throughout the life-cycle of materials, with respect to economic efficiency and social equity. The challenge is to overcome the barriers due to restraining forces, such as the speed of the material substitution process (quite low with respect to price volatility), the risks of performance losses, the technological locks due to investments and material qualification costs, the need for personnel education, etc.

## 5. Conclusions

In this survey, the role of critical elements, such as Co, W, Cr, Nb and Mg, in high value alloys and metal-matrix composites used under extreme conditions of temperature, wear, corrosion and loading was investigated. Some insights into a possible or already achieved reduction or substitution of these elements in advanced engineering materials are given. 

Profound understanding of the role of the CRMs on the evolution of the microstructure during the processing and service of high tech material and a careful evaluation of environmental and health impacts are key factors in materials’ substitution. Moreover, recent advances show that additive manufacturing constitutes a valid alternative for the reduction of CRMs in production processes.

The study carried out in this work shows how some of the analyzed critical elements are, at present, not substitutable for a few high extreme applications, while they are substitutable in less demanding ones. For instance, a complete elimination of Cr in stainless alloys at comparable corrosion performances of traditional 300-series alloys seems unfeasible. The only chance is to reduce the Cr amount by substituting it partially with Si and/or Al for applications at high temperatures and with Si and/or Mo in aqueous solutions. However, particular attention should be paid to Si use, also in consideration of the fact that Si was recently listed among the CRMs. Other chances to save Cr can be found by applying coatings (chemical conversion treatments, anti-wear and anticorrosion coatings, etc.) for further protection of stainless alloys containing reduced amounts of Cr.

On the contrary, substitution of WC-Co cemented carbides seems more feasible in most of the tool applications, apart from machining at very extreme loads. Ti-(C,N)-based cermets can be applied as cutting tools as an alternative for WC-Co cemented carbides. CRMs-free cermets and composites can provide good (not always comparable) performances at lower costs and have the advantages of being lightweight compared to WC-based hard metals. Moreover, for some applications, Co in cemented carbides may be partially or totally replaced by Ni-based binders without significant loss in performances. Also in this case, CRMs-free hard and super-hard coatings may be used to leverage the performances of other types of tool materials to be comparable with those of WC-Co tools.

Substitution of Co and Cr in Ni-based superalloys seems quite challenging due to the fundamental role of Cr and Co in conferring to the alloy oxidation resistance at high temperature and optimum tensile properties, yield strength and strain hardening capacity, respectively. Possible alternatives with reduced or no CRMs content are Ni-based ODS, Ti- and Fe-based intermetallics and ceramic matrix composites. The role of the microstructural design of intermetallic multi-phase γ-TiAl-based alloy for high temperature applications is worth mentioning. Also in Ni-based superalloys, CRM-free thermal barrier coatings may be used to further reduce CRM content and improve the high temperature strength of the alloy.

Ti, Ta and Mo seem potential candidates to substitute Nb in stainless steel, while Nb substitution in HSLA steels seems much more challenging. 

Finally, an alternative to Al-Mg alloys is represented by third generation Al-Li alloys. These Al alloys based on Cu and Li contain a much reduced Mg content at reduced weight and improved strength, toughness and corrosion resistance.
